# Autistic traits and suicidality in midlife and old age: investigating mediating effects of mental health and social connectedness

**DOI:** 10.1038/s44220-025-00579-0

**Published:** 2026-01-27

**Authors:** Eleanor Nuzum, Radvile Medeisyte, Aphrodite Eshetu, Sarah Hoare, Anne Corbett, Clive Ballard, Adam Hampshire, Elizabeth O’Nions, Amber John, Gavin R. Stewart, Joshua Stott

**Affiliations:** 1 UCL Research Department of Clinical, Educational and Health Psychology, Division of Psychology and Language Sciences, University College London, London, UK; 2 College of Medicine and Health, University of Exeter, Exeter, UK; 3 Neuroimaging Department, Institute of Psychiatry, Psychology and Neuroscience, King’s College London, London, UK; 4 Bradford Institute for Health Research, Bradford Centre for Health Data Science, Bradford Royal Infirmary, Bradford, UK; 5 Department of Psychology, University of Liverpool, Liverpool, UK; 6 Social Genetic & Developmental Psychiatry Centre, Institute of Psychiatry, Psychology and Neuroscience, King’s College London, London, UK

**Keywords:** Psychology, Risk factors

## Abstract

Suicidality is increased among middle-aged and older autistic adults, but little is known about the underlying factors linking autism with suicidality in midlife and older age. Here we report a cross-sectional observational study of 9,979 adults (76% female) aged 50+ years who completed questionnaires measuring autistic traits, current mental health, social connections and suicidality (suicidal ideation and suicidal self-harm). We use path analysis to explore the relationship between autistic traits and suicidality and the mediating effects of current mental health, social connectedness and male/female sex. Our results find that depression, anxiety, post-traumatic stress disorder (PTSD), loneliness and social isolation all significantly mediate the relationship between autistic traits and suicidal ideation, with small effect sizes. For suicidal self-harm, male sex, depression, PTSD and social isolation were found to be mediators. We conclude that mental health difficulties and social isolation mediate higher rates of suicidality in 50+-year-olds with high autistic traits. Targeted and individually tailored interventions for people on the autism spectrum across the lifespan are important.

## Abstract (web summary)

Nuzum et al. used cross-sectional data from the UK PROTECT study to evaluate the relationship between high autistic traits and suicidality in older adults.

## Main

Autism is a lifelong neurodevelopmental condition characterized by early onset social communication differences and repetitive and restrictive behaviors. Autism has an estimated global prevalence of 1% (ref. ^1^), with a 3:1 male-to-female ratio^2^. Epidemiological studies of autism prevalence have indicated high rates of underdiagnosis in adulthood, particularly in adults aged 50+ years, with approximately nine out of ten autistic people aged over 50 in the UK being potentially undiagnosed, or misdiagnosed with another condition^3,4^. So far, autism research has predominantly focused on children and young people, resulting in a scarcity of information about autism in midlife and older age^5^. Understanding the needs of aging autistic people is of great importance, as autistic adults have been found to have worse outcomes in terms of reduced life expectancy^6^, as well as a disproportionate risk of mental health difficulties such as anxiety, depression and periods of crises compared to non-autistic adults^7^.

One major public health concern is the high rate of suicidality and death by suicide reported in autistic populations. Suicide has been identified as the third leading cause of death for diagnosed autistic people (who are therefore a predominantly young group) in a Swedish population mortality study (accounting for 11.7% of deaths), with people diagnosed with autism being seven times more likely to die by suicide than non-autistic comparisons^8^. Suicidal ideation (SI; thoughts of death and life not being worth living) and suicidal self-harm (SSH; harming oneself with the intent to die) have also been reported at higher rates in autistic people/people with high autistic traits than in the general population^8,9^. Factors associated with suicidality have previously been examined in younger autistic populations, with depression, anxiety and experiences of abuse being identified as key risk factors^10–12^. However, despite these risk factors being elevated in middle-aged and older adults on the autism spectrum^9,13,14^, their relationship with suicidality has yet to be studied for this age group.

Loneliness (the subjective negative experience of aloneness) and social isolation (an objective lack of social contacts) have been associated with suicidality in the general population^15^. These negative social experiences are commonly experienced by autistic adults in midlife and older age^16,17^. However, the relationship between suicidality, loneliness, social isolation and autism has not been investigated in autistic people of any age.

Furthermore, although men (particularly those aged over 75 years^18^) are often found to be at the highest risk of death by suicide in the general population, autistic women have been found to be over-represented in autism suicide mortality studies^8^, suggesting that female sex is a risk factor for SSH in the autism population. However, the relationship between sex/gender, autism and suicide has not been investigated in middle-aged and older adults.

Given that suicide risk peaks in later life and that suicide is one of the leading causes of death in older age^18^, and those over 65 years are more likely to have experienced recent thoughts of suicide^19^, understanding the factors that influence suicide in aging autistic populations is a high-priority issue. Understanding the role of potential risk factors (for example, age, gender/sex, mental health difficulties, trauma, social isolation and loneliness) may help in developing clinical interventions and strategies for suicide risk assessment and management. As autism is often viewed as a spectrum of traits^20^, and due to the high rate of underdiagnosis in autistic adults aged 50+ years^3,4^, this study uses a trait-based approach to examine suicidality in a sample of middle-aged and older adults irrespective of autism diagnostic status. This trait-based approach to autism in underrecognized groups has been commonly used, with comparable profiles being found in those with high autistic traits and those with autism diagnoses^3^.

In this Article we explore the following: (1) whether any relationship between high autistic traits and suicidality (both SI and SSH) is mediated by current mental health symptoms including depression, anxiety, post-traumatic stress disorder (PTSD), loneliness and social isolation, and (2) whether female sex mediates the relationship between high autistic traits and SSH.

## Results

### Autistic trait group differences in suicidality

The high autistic spectrum traits (AST) group (*N* = 672), compared to the low AST group (*N* = 9,307), reported significantly higher rates of SI (experienced more than once, 29% versus 16%; *χ*^2^(2) = 58.78, *P* < 0.001) and SSH (6% versus 3%; *χ*^2^(1) = 13.82, *P* < 0.001) (Table 1).

**Table 1:** Ratings on the suicidality outcome variables across groups

Outcome	Frequency	Full sample	High AST	Low AST	Group difference
		*N* = 9,979	*N* = 672 (6.7%)	*N* = 9,307 (93.3%)	
**Suicidal ideation**	Never (*n*, %)	5,734 (70)	318 (58.1)^a^	5,416 (70.5)^a^	*χ*^2^(2) = 58.77, *P* < 0.001
	Once (*n*, %)	1,079 (13)	70 (12.8)	1,009 (13.1)	
	More than once (*n*, %)	1,419 (17)	159 (29.1)^a^	1,260 (16.4)^a^	
**Suicidal self-harm**	No (*n*, %)	7,991 (97)	515 (94.0)^a^	7,476 (97.3)^a^	*χ*^2^(1) = 13.82, *P* < 0.001
	Yes (*n*, %)	266 (3)	33 (6.0)^a^	233 (2.7)^a^	

Group difference is the difference in suicidality scores between high and low AST groups.^a^Significant difference in adjusted residual values between cells.

### Direct paths from autistic trait groups to suicidality

The direct and indirect path model (AST groups to SI/SSH through mediators) was found to have a good fit (*N* = 9,979, *χ*^2^(6) = 291.95, *P* < 0.001; comparative fit index = 0.98; Tucker Lewis index = 0.88; root-mean-square error of approximation = 0.069). In the full model, the direct path between AST group and SI (*β* = 0.04, s.e. = 0.03 *P* = 0.174) and SSH (*β* = 0.01,s.e. = 0.01 *P* = 0.076) were not statistically significant, suggesting that the differences between the high/low AST groups in SI and SSH were being mediated by indirect factors (Table 2).

**Table 2:** Scores on mental health and social isolation variables across groups

Measure	Statistic	Full sample	High AST	Low AST	Group difference
		*N* = 9,979	*N* = 672 (6.7%)	*N* = 9,307 (93.3%)	
**Depression** (scores = 0–24)	*M* (s.d.)	2.52 (3.23)	4.06 (4.71)	2.41 (3.06)	*t*(576.03) = −8.01, *P* < 0.001
**Anxiety** (scores = 0–24)	*M* (s.d.)	1.54 (2.69)	2.49 (3.87)	1.47 (2.57)	*t*(583) = −6.10, *P* < 0.001
**PTSD** (scores = 0–21)	*M* (s.d.)	1.19 (2.33)	1.79 (2.99)	1.14 (2.27)	*t*(515.73) = −4.60, *P* < 0.001
**Loneliness** (scores = 0–6)	*M* (s.d.)	4.20 (1.54)	5.05 (1.86)	4.14 (1.50)	*t*(735.38) = −1.06, *P* < 0.001
**Social isolation** (scores = 0–30)	*M* (s.d.)	13.02 (5.26)	16.40 (5.35)	12.78 (5.16)	*t*(764.09) = −16.98, *P* < 0.001

*M*, mean; s.d., standard deviation. Higher scores indicate more severe symptoms/greater social isolation. Group difference is the difference in scores between high and low AST groups.

### Indirect paths through current mental health, social connectedness and sex

High AST group membership was significantly associated with symptoms of depression (*β* = 1.60, s.e. = 0.14, *P* < 0.001), anxiety (*β* = 1.00, s.e. = 0.12*, P* < 0.001), PTSD (*β* = 0.61, s.e. = 0.11, *P* < 0.001), loneliness (*β* = 0.91, s.e. = 0.06, *P* < 0.001), social isolation (*β* = 3.62, s.e. = 0.21, *P* < 0.001) and male sex (*β* = −0.30, s.e. = 0.02, *P* < 0.001), all with individual large effect sizes.

**Fig. 1:**
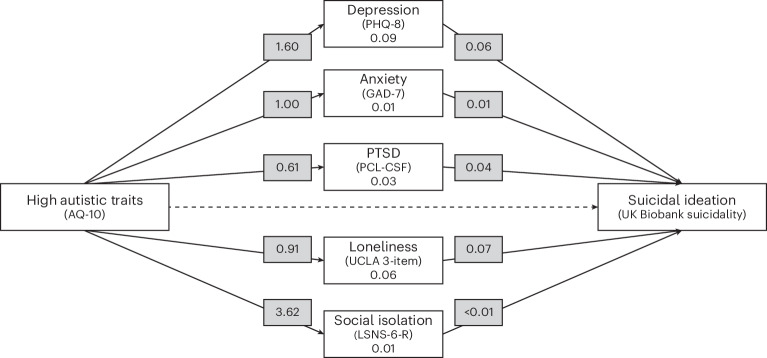
Path model of direct and indirect paths from high autistic traits to SI. Solid lines indicate significant associations, and dotted lines indicate non-significant associations. The numbers given are effect sizes (standardized estimates). The measures used are described in Methods.

SI was significantly associated with depression (*β* = 0.06, s.e. < 0.01, *P* < 0.001), anxiety (*β* = 0.01, s.e. < 0.01, *P* < 0.05), PTSD (*β* = 0.04, s.e. < 0.01, *P* < 0.001), loneliness (*β* = 0.07, s.e. < 0.01, *P* < 0.001) and social isolation (*β* < 0.01, s.e. < 0.01, *P* < 0.05). Sex was not included in this model due to saturation of pathways. The relationship between high AST group membership and SI was partially mediated by depression (*β* = 0.09, s.e. = 0.01, *P* < 0.001), anxiety (*β* = 0.01, s.e. < 0.01, *P* < 0.05), PTSD (*β* = 0.03, s.e. < 0.01, *P* < 0.001), loneliness (*β* = 0.06, s.e. < 0.01, *P* < 0.001) and social isolation (*β* = 0.01, s.e. < 0.01, *P* < 0.05), all with individual small effect sizes. The path analysis model is shown in Fig. 1.

**Fig. 2:**
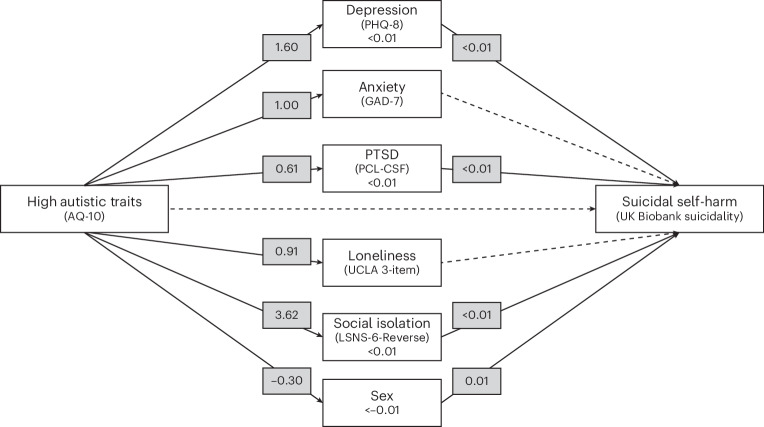
Path model of direct and indirect paths from high autistic traits to SSH. Solid lines indicate significant associations, and dotted lines indicate non-significant associations. The numbers given are effect sizes (standardized estimates). The measures used are described in Methods.

SSH was significantly associated with depression (*β* < 0.01, s.e. < 0.01, *P* < 0.001), PTSD (*β* < 0.01, s.e. = 0.001, *P* < 0.001), social isolation (*β* < 0.01, s.e. < 0.01, *P* < 0.001) and male sex (*β* = 0.01, s.e. < 0.01, *P* < 0.05), but not anxiety (*β* < −0.01, s.e. < 0.01 *P* = 0.24) or loneliness (*β* < 0.01, s.e. < 0.01, *P* = 0.84). This suggests that the relationship between high AST group membership and SSH was partially mediated by depression (*β* < 0.01, s.e. < 0.01, *P* < 0.001), PTSD (*β* = 0.05, s.e. < 0.01, *P* < 0.001), social isolation (*β* = 0.01, s.e. < 0.01, *P* < 0.001) and male sex (*β* < −0.01, s.e. < 0.01, *P* < 0.05), but not anxiety (*β* < −0.01, s.e. < 0.01 *P* < 0.50) or loneliness (*β* < 0.01, s.e. < 0.01, *P* < 0.50), all with individual small effect sizes. Figure 2 presents the path analysis model.

### Co-variance between mediators and outcomes

Depressive symptoms, anxiety symptoms, PTSD symptoms, loneliness and social isolation were all significantly associated with one another in the model (all *P* < 0.001) with large effect sizes. This would indicate that as one mediator increased in severity, so did the other mediators. SI and SSH were also significantly associated with each other (*β* = 0.03, s.e. < 0.01, *P* < 0.001), though this effect size was small.

## Discussion

This study reports on the risk factors that may underpin the relationship between suicidality and high autistic traits in adults aged 50+ years. We found significantly higher rates of SI and SSH in adults with high versus low autistic traits, with nearly one-third of those with high traits reporting lifetime SI. This relationship was fully mediated by a range of known risk factors, although individual effect sizes were small. Specifically, the autistic traits to SI direct path was mediated by symptoms of depression, anxiety, PTSD, feelings of loneliness and having few social contacts. The autistic traits to SSH direct path was mediated by symptoms of depression, PTSD, having few social contacts and male sex, but not symptoms of anxiety and feelings of loneliness. Although the individual effect sizes were small, the cumulative impact across multiple factors may be larger. These findings highlight that although middle-aged and older people on the autism spectrum may be at higher risk of SI and SSH than controls, rather than suicidality being an inherent feature of autism/autistic traits, suicidality is potentially a consequence of increased mental health problems and, to a lesser degree, social dislocation, in autistic populations. This has important implications for intervention.

Current depressive symptoms significantly mediated the relationship between high autistic traits and both SI and SSH. Depressive symptoms and diagnoses have been found to be reported at higher rates in high-autistic-trait adults aged 50+ years^13^. Given the well-established link between depression and suicide^21^, and depression being a major risk factor for suicidality among adults over 60 years^22^, these results align with research on the association between mental health symptoms and suicidality in younger autistic people^10^. Additionally, current symptoms of anxiety were also found to significantly mediate the relationship between high autistic traits and increased SI, but not SSH. The role of anxiety in suicidality remains unclear, particularly among autistic adults, with some studies reporting associations between anxiety disorders and SSH^23^, and others suggesting that anxiety is not an independent risk factor^24^. The present findings contribute to this ongoing debate, highlighting the need for further research into how anxiety influences suicidality in autistic individuals.

The symptoms of PTSD were found to be significantly higher in the high autistic trait group compared to the low autistic trait group—a finding comparable with the existing autism literature^9^. The role of PTSD symptoms as a mediator is consistent with previous research that has found associations between trauma exposure and experiences of suicidality in older adults^25^. Thus, the present study offers support to this evidence that PTSD symptoms, independent of depression and anxiety, are likely associated with both SI and SSH in middle-aged and older adults with high autistic traits. This has important clinical implications, highlighting the need for trauma-informed care and support for people on the autism spectrum in midlife and older age.

As per the findings of ref. ^16^, social isolation and loneliness were significantly more common in the high autistic trait group and were also mediators of suicidality. Loneliness and fewer social contacts mediated the relationship between high autistic traits and SI, with only social isolation being a significant mediator for SSH. Although not within the context of suicidality, our findings are complementary to those of ref. ^26^, which reported that robust social support is positively associated with quality of life for middle-aged and older autistic adults. Given that both loneliness and social isolation independently mediated the relationship between autistic traits and suicidality, our work highlights their importance (particularly social isolation, given the association with both ideation and self-harm) as potential targets for intervention. Additionally, it is important to consider both the quality and quantity of social relationships as distinct but interconnected factors in reducing suicide risk in autistic populations.

Finally, despite autistic women often having higher rates of SSH than autistic men, the current study found that male sex significantly mediated the relationship between high autistic traits and SSH, which was inconsistent with previous research on Swedish national records^8^, where female gender in autistic adults was associated with increased SSH. However, an important consideration is that the current study involves self-reported experiences from living participants engaging in voluntary research, rather than the examination of health records, which include those who have died by suicide. Additionally, given the relatively low endorsement of SSH in the current sample, it is important to consider how these sampling and response type effects may influence the influence of gender in the current study.

There are several strengths and limitations of this study. The study addresses the unique roles mental health and social problems play in the relationship between suicidality and autistic traits in middle-aged and older adults. Given the rarity of diagnosis in older people^4^, using trait-based measures is a strength in that it provides a more inclusive view of autistic traits and suicidality given that most measures of autism are biased to identify male-typical presentations of autism. However, although they have high traits, it is not possible to confirm that the identified sample would meet autism diagnostic criteria. Although the PROTECT dataset did include information on formal autism diagnoses, very few participants in these data had a confirmed diagnosis (for example, in a previous paper published using data from PROTECT, only 21 (0.1%) of 20,220 participants had an autism diagnosis^13^). As such, it was not possible to use this group in a statistical analysis.

Although the PROTECT cohort is predominantly female (>70%), we identified proportionately more men with high autistic traits than women, so the high AST group included comparable numbers of males and females, suggesting that the AQ-10 provided a relatively gender-inclusive measure of autistic traits in this sample, which is consistent with recent meta-analytic prevalence estimates^1^.

This was a cross-sectional observational study, which limits the ability to make causal links directly between autistic traits, mental health and suicidality. Although the results were significant, the small individual effect sizes may limit the degree to which these results can be applied to clinical practice. There was also a lack of diversity: the vast majority of participants were white and had a high educational level. The lack of representation of minoritized ethnic groups and diverse gender identities limits the generalizability of the findings to the general population and is a widespread issue in autism research^27,28^. There is also substantial evidence about the link between minority stress and suicidality^29,30^, so it is likely that having multiple minoritized identities (for example, being non-white, a gender minority, autistic, older) may substantially increase the risk of suicidality, and this warrants attention in future research.

Although the high- and low AST groups differed on some demographic characteristics (age, marital status and employment), these were not controlled for in the present analyses. Employment status may be less informative in this older sample, where retirement is common. However, future research should examine how demographic factors such as age and marital status influence the relationship between autistic traits, mental health and suicidality.

Finally, the measure of suicidality used in this study, which relies on self-report, has been widely used by the UK Biobank and has strong polygenic associations with people who died by suicide^31^. However, a key limitation of this measure was the use of very brief questions about SI and SSH, and in the present study only living people were asked these questions, so we cannot account for the people who have high autistic traits/autistic people who have died by suicide. The measure asked about suicidality in a person’s lifetime, including both current and earlier risk, whereas some of the risk factors in the analysis were current mental health symptoms. As such, the link between current mental health and current suicidality should be interpreted with caution, and further research is needed to clarify these temporal relationships.

Despite these limitations, there are key clinical implications to be considered as a result of this research. First, middle-aged and older adults who have high autistic traits are at an increased risk of poor mental health and social problems when compared to their peers with low autistic traits. As such, support that is tailored specifically to the needs of middle-aged and older people on the autism spectrum, and active outreach to middle-aged and older adults, is imperative for providing appropriate care to promote good mental health. Second, middle-aged and older adults with high autistic traits and poor mental health were more likely to experience both SI and SSH, indicating that middle-aged and older autistic adults may be more likely to act on suicidal thoughts in the form of SSH. This is of particular concern, as suicide rates among the older age groups are rising, and those with autistic traits appear to be at a heightened risk. Recent studies have highlighted the barriers to appropriate healthcare access that autistic people face^32,33^, including for autistic middle-aged and older women^34^ and middle-aged and older autistic adults who have experienced lifetime stressors^11^. Furthermore, autistic adults experiencing suicidality were recently found to not seek out public healthcare for support due to perceived barriers and lack of access to appropriate care^35^, highlighting the need for appropriate and timely help and support to mitigate this risk of reaching periods of crisis.

In conclusion, the present study has both affirmed the existing research that middle-aged and older adults with autistic traits are likely to be at increased risk of suicidality than the general middle-aged and older adult population, as well as highlighted why this may be the case by exploring the mediating role of current mental health and social connectedness. Taken together, this research highlights the growing need for middle-aged and older adults to continue to be included in autism research so as to understand this relationship with greater clarity. Interventions to support autistic adults aged over 50 years in their mental health and social support are crucially needed, and action is needed to adapt and tailor intervention to meet the needs of middle-aged older autistic adults to provide an effective service.

## Methods

### Study design

This study uses cross-sectional data from the PROTECT study, a UK-wide research study launched in 2015^36^ (www.protectstudy.org.uk). Participants complete annual online questionnaires centered around lifestyle, health and cognitive tests. Participants were recruited to the study via adverts in charities, the press and social media. Inclusion criteria for the PROTECT study were adults aged over 50 years, resident in the UK, with a good understanding of English, and able to use a computer with internet access. Only participants who had an established diagnosis of dementia at baseline were excluded (other psychiatric diagnoses were not used as exclusionary criteria). All participants gave written, informed consent. The PROTECT study received ethical approval from the UK London Bridge National Research Ethics Committee (ref. 13/LO/1578).

### Participants

From a total sample of over 20,000 PROTECT participants, 9,979 participants (75% female) aged 50–97 years had complete data and were included in the current study. Consistent with the literature highlighting up to 90% underdiagnosis of autism in middle-aged and older adults^3^, we operationalized autistic traits using a trait-based measure rather than relying on diagnosis. Using the standard cutoff of ≥6 on the Autism Spectrum Quotient 10-item scale (AQ-10), 672 (6.7%) of the participants were identified as having high autistic traits (the high AST group), with the remaining 9,307 participants forming a low autistic traits (low AST) group. Some differences were observed between groups; notably, members of the high AST group were older (mean age = 68.2 years versus 67.0 years) and more often male (48% versus 20%) than in the low AST group. Groups were broadly similar in ethnicity (90% white) and highest educational attainment (<62% with university-level qualifications) (Table 3).

**Table 3:** Descriptive statistics across the samples

Variable		Full sample	High AST	Low AST	Group difference
		*N* = 9,979	*N* = 672 (6.7%)	*N* = 9,307 (93.3%)	
Sex	Male/female	2,223:7,017 (22.3%:70.3%)	325:298 (48.4%:44.3%)	1,898:6,719 (20.4%:72.2%)	*χ*^2^(1) = 287.24, *P* < 0.001***
Age (years)	*M* (s.d.)	67.12 (7.17)	68.22 (7.86)	67.04 (7.12)	*t*(692.27) = −3.61, *P* < 0.001***
	Range	50–97	50–92	50–97	
Ethnicity	White	9,069	608 (90.5%)	8,461 (90.9%)	*χ*^2^(17) = 20.35, *P* = 0.260
	Mixed	60	4 (0.6%)	56 (0.6%)	
	Black	10	–	10 (0.1%)	
	Asian	75	10 (1.5%)	65 (0.7%)	
	Other	26	1 (0.1%)	25 (0.3%)	
Marital status	Married	6,232	405 (60.3%)^a^	5,827 (62.6%)^a^	*χ*^2^(6) = 20.05, *P* = 0.003**
	Widowed	706	45 (6.7%)	661 (7.1%)	
	Separated	133	8 (1.2%)	125 (1.3%)	
	Divorced	1,010	57 (8.5%)^a^	953 (10.2%)^a^	
	Civil partnership	51	1 (0.1%)	50 (0.5%)	
	Co-habiting	519	48 (7.1%)^a^	471 (5.1%)^a^	
	Single	587	59 (8.8%)^a^	528 (5.7%)^a^	
Education	School to 16	1,092	71 (10.6%)	1,021 (11.0%)	*χ*^2^(5) = 7.29, *P* = 0.200
	School to 18	2,822	190 (28.3%)	2,632 (28.3%)	
	Undergraduate	3,171	215 (32.0%)	2,956 (31.8%)	
	Postgraduate	2,155	147 (21.9%)	2,008 (21.6%)	
Current employment	Employed	3,315	185 (27.5%)^a^	3,130 (33.6%)^a^	*χ*^2^(4) = 23.612, *P* < 0.001***
	Retired	5,718	414 (61.6%)^a^	5,304 (57.0%)^a^	
	Unemployed	200	22 (3.3%)^a^	178 (1.9%)^a^	
Current voluntary work	Yes/no	4,393:4,774 (44.0%:47.8%)	304:314 (45.2%:46.7%)	4,089:4,460 (43.9%:47.9%)	*χ*^2^(5) = 3.19, *P* = 0.67

*M* , mean; s.d., standard deviation. Specific ethnicities were collapsed to create broader groups. *P < 0.05, **P < 0.001, ***P < 0.001. ^a^Significant difference in adjusted residual values between cells.

### Measures

Demographic information was collected using PROTECT’s online survey platform, including age, sex, marital status, education history and employment status.

Autistic traits were measured using the Autism Spectrum Quotient 10-item scale (AQ-10 (ref. ^37^). Its scores range from 0–10 (low to high traits), with a cutoff score of ≥6 being used for probable autism. This was therefore used in the present study to identify the high AST group, and those with a score below six were allocated to the low AST group. The AQ-10 has a sensitivity of 0.88, specificity of 0.91 and a positive predictive value of 0.85 for correctly identifying those with autism^37^.

SI and SSH were measured using the UK Biobank Self-harm and Suicidality questionnaire^31^. SI was measured by asking participants to respond ‘never/once/more than once’ if they ‘have thought life was not worth living’. SSH was measured by asking participants to respond ‘yes/no’ if they ‘have harmed themselves with the intention of ending their lives’. Both questions also had a ‘prefer not to say’ response option, which was subsequently treated as missing data. This measure has previously been used widely in this way, including in studies examining suicidality in middle-aged and older adults with high autistic traits^9^.

Symptoms of recent depression were measured using the Patient Health Questionnaire (PHQ-8 (ref. ^38^). The PHQ-8 is an eight-item questionnaire (rated on a four-point scale, maximum score = 24) examining low mood over the past two weeks. The PHQ-8 is adapted from the original nine-item PHQ, with omission of the self-harm and suicidality item. The PHQ-8 was used to avoid item overlap with the suicidality questionnaire. The PHQ-8 has excellent psychometric properties, with a cutoff score of ≥10 having 88% sensitivity and 88% specificity for major depressive disorder^38^. The original nine-item PHQ has been found to have good psychometric properties for assessing depression symptoms in autistic populations^10^.

Symptoms of current anxiety were measured using the General Anxiety Disorder questionnaire^39^. The GAD-7 is a seven-item questionnaire (rated on a four-point scale, maximum score = 24) examining nervousness, uncontrollability of worrying, issues relaxing, restlessness and irritability. The GAD-7 has excellent psychometric properties, with a cutoff score of ≥10 having 89% sensitivity and 82% specificity for generalized anxiety disorder. Although not formally validated for use in autistic populations, the GAD-7 has been found to have very good psychometric properties in autistic adults (for example, Cronbach’s alpha = 0.78 (ref. ^16^).

Symptoms of current post-traumatic stress were measured using the PTSD Checklist–Civilian Short Form (PCL-CSF^40^). The PCL-CSF is a five-item questionnaire (rated on a four-point scale, maximum score = 21) examining avoidant behaviors and repeated intrusive thoughts and memories. The PCL-CSF has been found to be a reliable measure, with an internal consistency above 0.75 (ref. ^40^).

Feelings of current loneliness were measured using the UCLA three-item Loneliness Scale (UCLA-3LS^41^). The UCLA-3LS is a three-item questionnaire (rated on a three-point scale, maximum score = 9) examining subjective dissatisfaction with social relationships, with higher scores indicating loneliness. The UCLA-3LS has been found to have good internal consistency and reliability (Cronbach’s alpha = 0.72 (ref. ^41^) and the UCLA 20-item version is the preferred loneliness measure among autistic adults^42^.

Social isolation, or poor social connectedness, was measured using the Lubben Social Networking Scale (LSNS-6 (ref. ^43^). The LSNS-6 is a six-item questionnaire (rated on a six-point scale (‘zero’ to ‘nine or more’ social contacts), maximum score = 30) examining the frequency and quality of contact with family members and friends. The LSNS-6 has been shown to have high levels of internal consistency, stable factor structures and high correlations with criterion variables^43^. It has good internal consistency in autistic adults (Cronbach’s alpha = 0.83 (ref. ^16^). The measure was reverse-scored for consistency with other variables, with higher values indicating fewer social contacts—that is, greater social isolation.

### Statistical analysis

Analyses were conducted using RStudio (v4.31). Data were cleaned before analysis (Supplementary Information). Statistical power was considered good as per various guidelines for structural equation modeling (SEM^44,45^). Missing data were handled using full information maximum likelihood (the Supplementary Information provides details on missing data). *χ*^2^ analyses examined group differences (high/low AST) in suicidality measures (SI/SSH). The path model approach was chosen based on theory and previous research subject to good fit indices, and alternate models were not tested, as the focus was on evaluating this theory-driven model. SEM, specifically path modeling, examined whether the relationship between AST group and SI (0–2)/SSH (0–1) was mediated by current symptoms of depression, anxiety and PTSD, feelings of loneliness, social isolation and sex (male/female). The direct path (AST group to SI/SSH) and indirect paths (AST group to SI/SSH through mediators) were modeled using the ‘lavaan’ package add-on. To determine effect sizes, Cohen’s^46^ guidelines were used: a standardized regression value (*β*) between 0.10 and 0.29 was interpreted as a small effect size, *β* between 0.30 and 0.49 as a medium effect size, and *β* of 0.50 or greater as a large effect size.

### Reporting summary

Further information on research design is available in the Nature Portfolio Reporting Summary linked to this Article.

## Supplementary information

Supplementary Information Data cleaning summary and Supplementary Table 1.

Reporting Summary

## Data Availability

The data that support the findings of this study are held by the PROTECT team, but the availability of these data is restricted. The data were used under license to J.S. for the current study and are not publicly available. However, the data may be available from the authors upon reasonable request and with permission from J.S. and the PROTECT team.

## Code availability

All data analyses used readily available programs such as open-source R code. No custom code was used.
