# Supplementary material for: Autistic traits and suicidality in midlife and old age: investigating mediating effects of mental health and social connectedness
Source: Nat Ment Health. 2026 Jan 27;4(2):255–62. doi: 10.1038/s44220-025-00579-0 (PMC12890584; doi:10.1038/s44220-025-00579-0)
Supplement: Supplementary file 1 — Data cleaning summary and Supplementary Table 1. [file 44220_2025_579_MOESM1_ESM.pdf]

nature mental health

Supplementary information https://doi.org/10.1038/s44220-025-00579-0

Autistic traits and suicidality in midlife and old age: investigating mediating effects of mental health and social connectedness

In the format provided by the authors and unedited

<u>Supplementary Material 1</u>

Data Cleaning. The variables used in the analyses were thoroughly cleaned prior to analyses. This involved checking for missing values and identifying 'prefer not to say' and 'do not know' responses to ensure that different types of missing data were correctly identified. Missing data can be defined as 'missing completely at random' (MCAR), 'missing at random' (MAR) or 'not missing at random' (NMAR). In order to assess missing data, statistical tests (t-tests and chi-squares) were performed to compare the demographic profile between those with complete data and those with missing data, as well as checking for any significant differences in AQ-10 score between those with missing and complete data. Little's (1988) test for whether data was MCAR was also performed. In order to manage the missing data in a way that avoided deleting a large proportion of participants or imputing data, Full Information Maximum Likelihood (FIML) was used in the analyses, whereby if a participant had missing items on a variable, they were excluded from that part of the analyses but were still included in other parts of the analyses where their data was non-missing as is recommended when data are MAR as well as MCAR (Newsom, 2018).

Data were thoroughly cleaned, and missing data were dealt with using fiml which is robust to missing data missing completely at random (MCAR) or missing at random).

Demographics, current mental health variables and suicidality variables were extracted from the same timepoint that the first AQ-10 was completed to ensure that each participant had a profile of responses from one timepoint only.

Supplementary Material 2

Supplementary Table 1. Distribution of full list of ethnicities

Ethnicity	n (%)
White: English/Welsh/Scottish/Northern Irish/British	8,586 (93%)
White: Irish	120 (1%)
White: Gypsy or Irish Traveller	1 (<0.1%)
White: European	270 (3%)
White: Non-European	92 (<1%)
Mixed: White and Black Caribbean	8 (<0.1%)
Mixed: White and Black African	8 (<0.1%)
Mixed: White and Asian	24 (<1%)
Mixed: Any other mixed/multiple ethnic background	20 (<1%)
Asian/Asian British: Indian	35 (<1%)
Asian/Asian British: Pakistani	6 (<0.1%)
Asian/Asian British: Bangladeshi	1 (<0.1%)
Asian/Asian British: Chinese	19 (<1%)
Asian/Asian British: Any other Asian Background	14 (<1%)
Black/Black British: African	1 (<0.1%)
Black/Black British: Caribbean	9 (<1%)
Any other Black/African/Caribbean background	0 (0%)
Other ethnic group: Arab	2 (<0.1%)
Any other ethnic group	24 (<1%)
