# Supplementary material for: Autistic traits and suicidality in midlife and old age: investigating mediating effects of mental health and social connectedness
Source: Nat Ment Health. 2026 Jan 27;4(2):255–62. doi: 10.1038/s44220-025-00579-0 (PMC12890584; doi:10.1038/s44220-025-00579-0)
Supplement: Supplementary file 2 — Reporting Summary [file 44220_2025_579_MOESM2_ESM.pdf]

nature portfolio

Corresponding author(s): Professor Josh Stott

Last updated by author(s): 10/10/2025

Reporting Summary

Nature Portfolio wishes to improve the reproducibility of the work that we publish. This form provides structure for consistency and transparency in reporting. For further information on Nature Portfolio policies, see our Editorial Policies and the Editorial Policy Checklist.

Statistics

For all statistical analyses, confirm that the following items are present in the figure legend, table legend, main text, or Methods section.

n/a	Confirmed	
☐	☒	The exact sample size (*n*) for each experimental group/condition, given as a discrete number and unit of measurement
☐	☒	A statement on whether measurements were taken from distinct samples or whether the same sample was measured repeatedly
☐	☒	The statistical test(s) used AND whether they are one- or two-sided<br>*Only common tests should be described solely by name; describe more complex techniques in the Methods section.*
☐	☒	A description of all covariates tested
☐	☒	A description of any assumptions or corrections, such as tests of normality and adjustment for multiple comparisons
☐	☒	A full description of the statistical parameters including central tendency (e.g. means) or other basic estimates (e.g. regression coefficient) AND variation (e.g. standard deviation) or associated estimates of uncertainty (e.g. confidence intervals)
☐	☒	For null hypothesis testing, the test statistic (e.g. *F*, *t*, *r*) with confidence intervals, effect sizes, degrees of freedom and *P* value noted<br>*Give P values as exact values whenever suitable.*
☒	☐	For Bayesian analysis, information on the choice of priors and Markov chain Monte Carlo settings
☐	☒	For hierarchical and complex designs, identification of the appropriate level for tests and full reporting of outcomes
☐	☒	Estimates of effect sizes (e.g. Cohen's *d*, Pearson's *r*), indicating how they were calculated

Our web collection on statistics for biologists contains articles on many of the points above.

Software and code

Policy information about availability of computer code

Data collection	Data were previously collected by the PROTECT team and made available under license to Prof Josh Stott for the present study to engage in data analyses.
Data analysis	Analyses were conducted using RStudio (v4.31).

For manuscripts utilizing custom algorithms or software that are central to the research but not yet described in published literature, software must be made available to editors and reviewers. We strongly encourage code deposition in a community repository (e.g. GitHub). See the Nature Portfolio guidelines for submitting code & software for further information.

Data

Policy information about availability of data

All manuscripts must include a data availability statement. This statement should provide the following information, where applicable:

- Accession codes, unique identifiers, or web links for publicly available datasets
- A description of any restrictions on data availability
- For clinical datasets or third party data, please ensure that the statement adheres to our policy

The data that support the findings of this study are held by the PROTECT team, but the availability of these data is restricted. The data were used under license to Prof Josh Stott for the current study and are not publicly available. However, the data may be available from the authors upon reasonable request and with permission from Prof Josh Stott (j.stott@ucl.ac.uk) and the PROTECT team.

Research involving human participants, their data, or biological material

Policy information about studies with human participants or human data. See also policy information about sex, gender (identity/presentation), and sexual orientation and race, ethnicity and racism.

Reporting on sex and gender	Sex was recorded and reported as sex assigned at birth: male or female
Reporting on race, ethnicity, or other socially relevant groupings	Ethnicity was comprised of 19 groups to select from, where the groups broadly came under the umbrellas 'White', 'Mixed', 'Asian', 'Black' and 'Other'. The full list of ethnicities can be found in the appendices.
Population characteristics	Age, sex assigned at birth, ethnicity, marital status, education level, employment status and volunteering activity were recorded from the full sample and included in the dataset. Age was recorded based on the age the participant was at the time of completing the Autism questionnaires. There was no data available for gender identity, so sex assigned at birth (male/female) was used. Marital status comprised of 7 options: 'Married', 'Widowed', 'Separated', 'Divorced', 'Civil Partnership', 'Co-habitating' and 'Single'. Education level was recorded as the highest education level that had been reached at the time of completing the Autism questionnaires. Participants selected from: 'Secondary Education', 'Post-Secondary Education', 'Vocational Qualification', 'Undergraduate Degree', 'Post-Graduate Degree' and 'Doctorate'. For simplicity of reporting, these were then grouped as 'School to 16 (secondary education)', 'School to 18 (post-secondary education & vocational qualification)','Undergraduate' and 'Postgraduate (including doctorate)'. Employment status was recorded as participant's current employment status at the time of completing the Autism questionnaire. Participants selected from 'Employed full-time', 'Employed part-time', 'Self-employed', 'Retired' and 'Unemployed' which were then condensed into three categories: 'Employed', 'Retired' and 'Unemployed'. Volunteering work was recorded as the current number of hours participants were engaging in voluntary work at the time of completing the autism questionnaire and this information was condensed into whether they were or were not currently engaged in voluntary work.
Recruitment	Participants were part of the wider PROTECT study (www.protectstudy.org.uk) which is a UK-wide research study that was launched in 2015 aimed at understanding brain changes and the underlying causes of dementia in middle and later adulthood (Corbett et al., 2023). Participants completed annual questionnaires centred around lifestyle, health and cognition. The initial PROTECT sample consisted of 20,200 adults aged over 50 years. Participants were recruited to the study via advertising from Alzheimer's Society UK, the UK Medical Research Council, press coverage by the British Broadcasting Corporation (BBC), and on social media. Inclusion criteria for the PROTECT study were adults aged over 50 years, resident in the United Kingdom, with a good understanding of English, and able to use a computer with internet access. Participants who had an established diagnosis of dementia at baseline were excluded. Eligible participants registered online and were required to read an information sheet before providing consent to take part. No additional recruitment took place for the purpose of the present study as the relevant questionnaires were already embedded into the existing PROTECT dataset. Further details of the PROTECT study can be found at http://www.protectstudy.org.uk.
Ethics oversight	The PROTECT study received ethical approval from the U.K. London Bridge National Research Ethics Committee (Ref: 13/LO/1578).

Note that full information on the approval of the study protocol must also be provided in the manuscript.

Field-specific reporting

Please select the one below that is the best fit for your research. If you are not sure, read the appropriate sections before making your selection.

☒ Life sciences ☐ Behavioural & social sciences ☐ Ecological, evolutionary & environmental sciences

For a reference copy of the document with all sections, see nature.com/documents/nr-reporting-summary-flat.pdf

Life sciences study design

All studies must disclose on these points even when the disclosure is negative.

Sample size	Sample size is a complex issue in SEM without widely agreed upon methodologies. Two approaches were used to estimate the sample size required for this study. Fritz & MacKinnon (2007) surveyed the literature to examine the median sample sizes used for mediation analyses using SEM and found this to be 340.5 participants. Additionally, the widely used rule of thumb approach of 10 cases to 1 indicator variable was considered (Nunnally, 1967). In both cases, the sample size in the PROTECT study was in excess of what was required.
Data exclusions	No data was excluded. Full Information Maximum Likelihood was used to handle any missing data.
Replication	Replication was not undertaken as part of this study. The analyses were conducted using a theory-driven, a priori specified model consistent with the study's conceptual framework.
Randomization	Randomisation did not take place for this study. Participants were assigned high and low AST groups based on their scores on the AQ-10 questionnaire.
Blinding	No blinding was used for this study.

Reporting for specific materials, systems and methods

We require information from authors about some types of materials, experimental systems and methods used in many studies. Here, indicate whether each material, system or method listed is relevant to your study. If you are not sure if a list item applies to your research, read the appropriate section before selecting a response.

Materials & experimental systems

n/a	Involved in the study
☒	☐ Antibodies
☒	☐ Eukaryotic cell lines
☒	☐ Palaeontology and archaeology
☒	☐ Animals and other organisms
☒	☐ Clinical data
☒	☐ Dual use research of concern
☒	☐ Plants

Methods

n/a	Involved in the study
☒	☐ ChIP-seq
☒	☐ Flow cytometry
☒	☐ MRI-based neuroimaging

Plants

Seed stocks

Report on the source of all seed stocks or other plant material used. If applicable, state the seed stock centre and catalogue number. If plant specimens were collected from the field, describe the collection location, date and sampling procedures.

Novel plant genotypes

Describe the methods by which all novel plant genotypes were produced. This includes those generated by transgenic approaches, gene editing, chemical/radiation-based mutagenesis and hybridization. For transgenic lines, describe the transformation method, the number of independent lines analyzed and the generation upon which experiments were performed. For gene-edited lines, describe the editor used, the endogenous sequence targeted for editing, the targeting guide RNA sequence (if applicable) and how the editor was applied.

Authentication

Describe any authentication procedures for each seed stock used or novel genotype generated. Describe any experiments used to assess the effect of a mutation and, where applicable, how potential secondary effects (e.g. second site T-DNA insertions, mosiacism, off-target gene editing) were examined.
